# Analysis of the causal relationship between immune cells and rheumatoid arthritis from the perspective of genetic variation: a bidirectional two-sample Mendelian randomization study

**DOI:** 10.1186/s42358-024-00425-4

**Published:** 2024-11-01

**Authors:** Feng Cheng, YingJia Zhu, XiaoQian Liu, RuiKun Zhang, Fei Xia, LinPu Ge

**Affiliations:** 1 Department of Orthopedics, The Third Affiliated Hospital of Zhejiang Chinese Medical University, Hangzhou, Zhejiang China; 2 Department of Gynecology, Hangzhou Women’s Hospital, Hangzhou, Zhejiang China; 3 Department of Endocrinology, Hangzhou Hospital of Traditional Chinese Medicine, Hangzhou, Zhejiang China; 4 Zhejiang Chinese Medical University, Hangzhou, Zhejiang China; 5 Department of Anesthesiology, The Third Affiliated Hospital of Zhejiang Chinese Medical University, Hangzhou, Zhejiang China

**Keywords:** Rheumatoid arthritis, Immune cells, Causal inference, Genetic variation, MR analysis, Sensitivity

## Abstract

**Background:**

Immune factors are crucial in the pathogenesis of rheumatoid arthritis (RA), and immune cells play a key role in the development of RA. However, there is still disagreement regarding the specific roles of each type of immune cell in the pathological process of RA.

**Methods:**

This study used bidirectional two-sample Mendelian randomization (MR) analysis to determine the causal relationship between immune cell characteristics and RA. Utilizing publicly available genetic data, we initially treated immune cell characteristics as exposures to investigate their causal effects on the risk of RA. Subsequently, we performed reverse two-sample MR using the positively selected cells from the initial analysis as outcomes, aiming to identify the core immune cells involved. Finally, a comprehensive sensitivity analysis was conducted to validate the robustness, heterogeneity, and horizontal pleiotropy of the results.

**Results:**

Using data from 731 immune cells as exposures and cell SNPs as instruments, we independently conducted two-sample MR analysis for each patient with RA. The main analytical method used was the IVW method, with a significance level set at *P* < 0.05 for inclusion. In total, we identified 42 immune cell phenotypes that were causally associated with the onset of RA. For the reverse MR analysis, we used RA as the exposure factor and focused on 42 immune cell phenotypes as outcomes. Our analysis revealed causal relationships between the onset of RA and 7 immune cell phenotypes. Among these, 6 showed positive causal relationships, while 1 exhibited a negative causal relationship.

**Conclusions:**

Our study emphasized the causal relationship between immune cells and RA through bidirectional two-sample MR analysis, identifying the immune cells causally associated with RA.

**Supplementary Information:**

The online version contains supplementary material available at 10.1186/s42358-024-00425-4.

## Introduction

Rheumatoid arthritis (RA) is a chronic inflammatory joint disease characterized by joint pain, swelling, and functional impairment [1]. According to the World Health Organization, millions of people worldwide are affected by rheumatoid arthritis, with a higher prevalence among women and older individuals [2]. The exact cause of RA is not fully understood, but it is thought to result from the interaction of genetic, environmental, and immune factors [3]. Due to diagnostic challenges, high treatment costs, and individual variations, the treatment of RA remains a global challenge. Currently, there is no cure for RA, and management primarily involves symptom relief and reduction of inflammation through medication, physical therapy, and lifestyle modifications [4]. The current focus of research is on finding new early diagnostic methods and novel therapeutic targets for drug treatment [5].

The correlation between immune cells and RA has long been a topic of debate. RA pathogenesis, diagnosis, and treatment are closely associated with immune factors, and there is a wealth of domestic and international research focusing on immune factors [6]. Immune cells play a critical role in immune functions, and there are many studies related to this phenomenon. Some studies have shown that the interaction between immune cells and fibroblasts is the basis for the imbalance between regulatory T cells and helper T 17 cells, which not only exacerbates inflammation but also exacerbates bone destruction [7]. Other studies have used many clinical samples to analyse 18 peripheral blood immune cell subgroups through RNA sequencing, systematically describing the transcriptome changes and treatment effects of RA, and evaluating the relationship between immune cell gene modules and treatment resistance [8]. The chemokine receptor CCR6 is expressed on various cells in RA, such as B cells, immature dendritic cells, innate lymphoid cells (ILCs), regulatory CD4+ T cells, and Th17 cells [9, 10]. Increasing amounts of evidence indicate that different types of immune cells can participate in the development of RA. However, there is still disagreement about the role of each type of immune cell in the pathological process of arthritis. Moreover, many pieces of evidence thus far have been based on observational studies and may be limited by confounding factors and reverse causality.

MR was originally developed as an alternative approach to randomized controlled trials (RCTs) for establishing causal relationships between exposures and outcomes using genetic variants [11]. Due to the random allocation of genetic variations during conception prior to disease onset, MR is considered superior to traditional RCT studies in identifying causal relationships by effectively controlling for confounding factors and obtaining more accurate conclusions [12]. RA is the result of interactions between genetic susceptibility, environmental factors, and immune factors, with genetic factors accounting for 50–60% of the risk for RA [13]. Therefore, many recent studies have used two-sample Mendelian randomization (TSMR) to investigate causal relationships between various exposure factors and RA outcomes. Some studies have explored causal relationships between diseases, such as the relationship between RA and skin cancer [14]. Other studies have focused on drug targets, such as by examining the causal relationship between antidiabetic drugs and RA to identify more effective drug targets [15]. Additionally, extensive research has been conducted on the gut microbiota and DNA methylation in relation to RA and MR. However, few studies have evaluated the causal relationship between circulating immune cell counts and the onset of RA, particularly those utilizing MR analysis with 731 immune cells as exposure factors. To date, such research remains scarce.

In this study, we conducted bidirectional two-sample Mendelian randomization analysis for the first time to evaluate the causal bidirectional relationship between immune characteristics and RA. The aim of this study was to increase the understanding of the connection between these two systems among researchers and to provide an appropriate framework for understanding how different immune factors affect the development of RA. These findings may also provide useful clues for future research on immune cell function. Additionally, the reverse MR method was used to explore the causal relationship between circulating immune cells and the incidence of RA, which could help to identify risk factors for immune cells related to RA progression. This will help develop new diagnostic and therapeutic methods and contribute to finding solutions for this global challenge.

## Materials and methods

### Study design

In this study, we conducted bidirectional MR analysis to evaluate the causal relationship between 731 immune cell characteristics and RA. First, we used immune cell characteristics as the exposure factor, SNPs of each immune cell as instrumental variables, and RA as the outcome. We performed a Mendelian randomization analysis for each immune cell and RA using the inverse-variance weighting (IVW) method as the primary statistical analysis and selected results with *P* < 0.05. Then, we conducted reverse Mendelian randomization analysis using RA as the exposure factor and immune cells with positive results as the outcome. Sensitivity analyses were performed for all the obtained results to ensure their reliability. Additionally, the instrumental variables (IVs) used in our causal inference needed to satisfy three key assumptions [16]: (1) genetic variation is directly associated with the exposure; (2) genetic variation is unrelated to potential confounders between the exposure and outcome; and (3) genetic variation does not affect the outcome through pathways other than the exposure. All data used in this analysis were obtained from publicly available online databases, and the studies included in our analysis were approved by relevant institutional review boards.

The relevant genome-wide association study (GWAS) dataset was obtained from the IEU OpenGWAS project (https://gwas.mrcieu.ac.uk). Detailed information about the data can be found in the original publication [17]. To ensure consistency with the exposure data, summary data for RA were collected from the IEU OpenGWAS project (ID: “ebi-a-GCST90018910”), which included 8255 cases and 409,001 European ancestry controls. The dataset comprised 24,175,266 SNPs, and the RA patients in the summary data met the 1987 American College of Rheumatology (ACR) diagnostic criteria for RA and were positive for autoantibodies. Principal component analysis using GWAS data from three independent studies was used to match controls to BRASS RA patients [18].

When considering RA as the exposure factor, three criteria were applied to select appropriate SNPs. First, SNPs that were significantly associated with RA with a p value below the genome-wide significance threshold (1 × 10^−8^) were chosen. Second, the independence between the selected SNPs was evaluated based on pairwise linkage disequilibrium [19]. SNPs that were in high linkage disequilibrium (r^2^ > 0.001) with other SNPs within a clumping window of 10,000 kb were removed, even if they were associated with the exposure or outcome with a higher p value. Third, the strength of each individual SNP was evaluated by calculating the F-statistic. SNPs with an F-statistic > 10 were considered sufficiently strong to reduce potential bias. Prior to conducting MR analysis, data harmonization was conducted to ensure that the SNPs influencing the exposure and outcome corresponded to the same allele.

### Immunity-wide GWAS data sources

According to the GWAS catalogue, the summary statistics of each immune trait can be obtained publicly (registration numbers ranging from GCST0001391 to GCST0002121) [20]. A total of 731 immune phenotypes were included, including absolute cell count (AC) (*n* = 118), median fluorescence intensity (MFI) reflecting surface antigen levels (*n* = 389), morphological parameters (MP) (*n* = 32), and relative cell count (RC) (*n* = 192). Specific proteins and molecules on the surfaces of immune cells can be used to differentiate various types of immune cells and can be used for the diagnosis and monitoring of immune system function. The four immune features of immune cells include surface markers, cell population counts, cell activity, and morphological parameters, all of which are necessary for evaluating the normal functioning of the immune system and are widely applied in immunological research and diagnosis. Specifically, MFI, AC, and RC features include B cells, CDCs, mature T cells, monocytes, bone marrow cells, TBNKs (T cells, B cells, natural killer cells), and Treg cells, while MP features include CDCs and TBNK cells. According to recent studies [21], when immune cells are considered exposure factors, we set the significance level of IVs for each immune trait to 1 × 10^−5^.

### Statistical analysis

We ensured that the effect of SNPs on exposure and outcome corresponded to the same alleles by harmonising the summary statistics for both datasets. To infer causal associations, we conducted TSMR analyses using multiple methods, including IVW, weighted median regression, MR‒Egger regression, simple mode, and weighted mode. The IVW method, as a primary approach for estimating the causal relationship between exposure and outcome, is used to calculate the ratio of the effect size of single-nucleotide polymorphisms (SNPs) associated with the outcome to the effect size of SNPs associated with the exposure [22]. The IVW method was used as the primary method for MR, which combined the Wald ratio estimates of different SNPs to provide a consistent estimate of the causal effect of exposure on the outcome [23]. The reliability of the IVW method depends on the absence of horizontal pleiotropy of the IVs [24]. When at least half of the SNPs were effective IVs, the weighted median method provided a consistent estimate of the causal effect [25]. MR‒Egger regression was used to confirm the existence of horizontal pleiotropy, and its intercept represented the effect estimate of horizontal pleiotropy [26]. Even when the IVs have horizontal pleiotropy, MR‒Egger regression can still be used to obtain an unbiased estimation of causal associations. Compared to the MR‒Egger method, the weighted median method improved the accuracy of the results [27]. Simple mode and weighted mode were used for complementary analyses [28]. Additionally, scatter plots and funnel plots were used to demonstrate the robustness of the correlation and lack of heterogeneity. All analyses were conducted in R 4.3.1 software using the R packages TwoSampleMR and MR-PRESSO [29]. The R packages randomForest and ggplot2 were used for plotting.

### Sensitivity analyses

Sensitivity analysis is crucial for ensuring the robustness of our conclusions. These analyses primarily involve heterogeneity testing to assess differences among instrumental variables (IVs). If significant differences exist among IVs, this indicates heterogeneity within these IVs. The commonly used test for this purpose is Cochran’s Q statistic, where *p* values greater than 0.05 for both MR‒Egger and IVW Cochran’s Q suggest no heterogeneity. Of particular importance is the assessment of horizontal pleiotropy, for which MR‒Egger regression is a commonly employed method that can be used to detect pleiotropic effects of genetic variation. By calculating the MR‒Egger intercept, researchers can assess whether all genetic effects are attributable to a single biological pathway. A P value greater than 0.05 for the MR‒Egger intercept indicates no horizontal pleiotropy. Other methods, such as leave-one-out (LOO) cross-validation and MR-pleiotropy residual sum and outlier (MR–PRESSO), are also utilized. LOO testing involves iteratively excluding individual observations from the analysis dataset to assess their individual impact on the results. MR–PRESSO is a statistical tool used to detect potential shared genetic effects (pleiotropy) influencing the genotype-outcome relationship [30].

## Results

### Investigating the causal relationship between immune cells and RA

To determine the relationship between immune cells and the development of RA, we utilized 731 data points of immune cells as the exposure and the effect on cell SNPs as instrumental variables to conduct two-sample MR analyses with RA. With the IVW method as the primary analytical approach and a significance threshold of *P* < 0.05, we identified a total of 42 immune cell phenotypes with causality in the development of RA, categorized into 7 major groups, including 6 B-cell types, 5 CDC types, 9 myeloid cell types, 6 maturation stages of T-cell types, 4 TBNK types, 5 Treg cell types, and 7 monocyte types. Specifically, among the trait types, the majority were MFIs, accounting for 30 types, followed by 8 RC types, 3 AC types, and only 1 MP type, which had the smallest sample size (Supplementary Table 1).

**Fig. 1:**
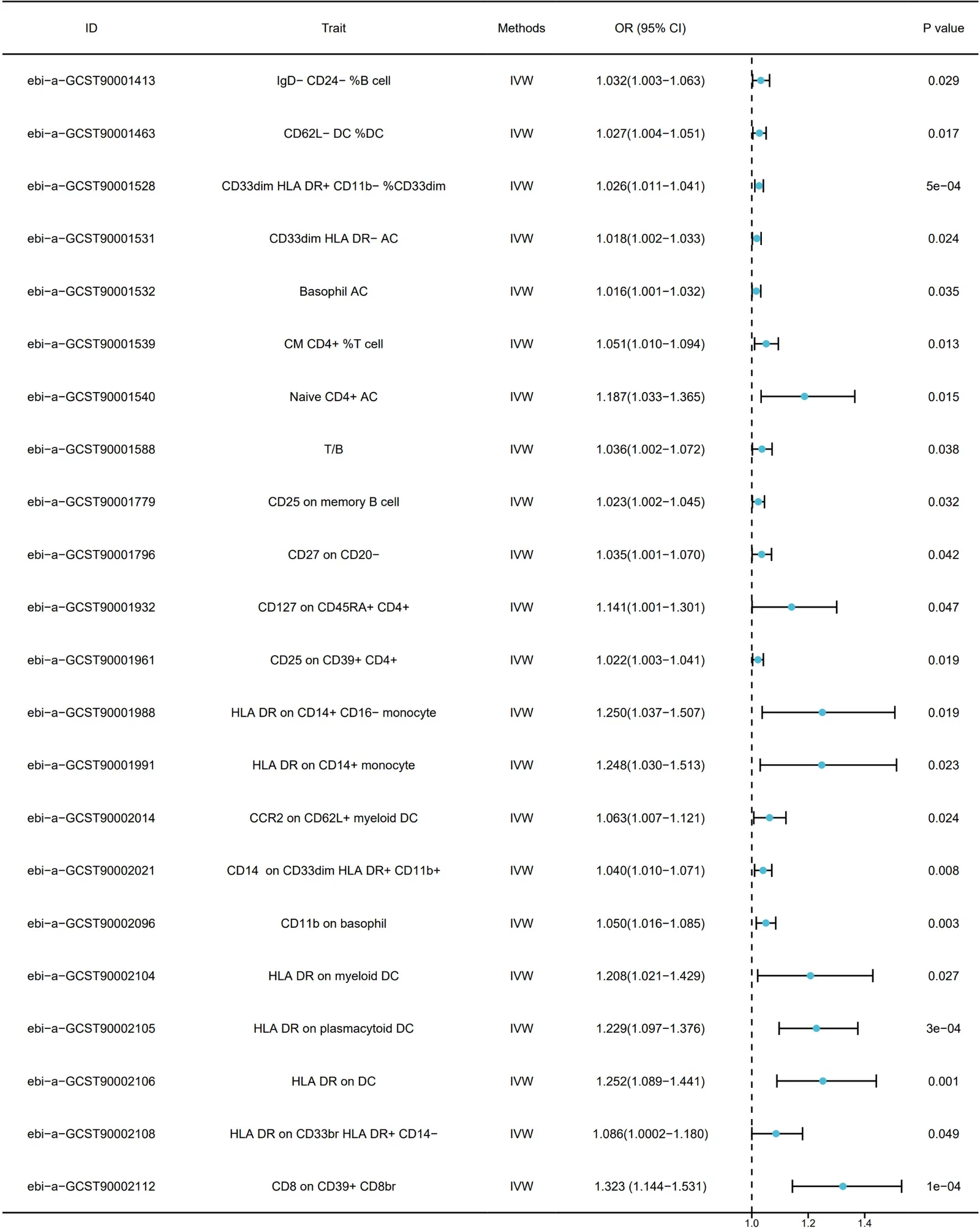
Forest plots showing the causal associations between RA and 22 immune cell traits

**Fig. 2:**
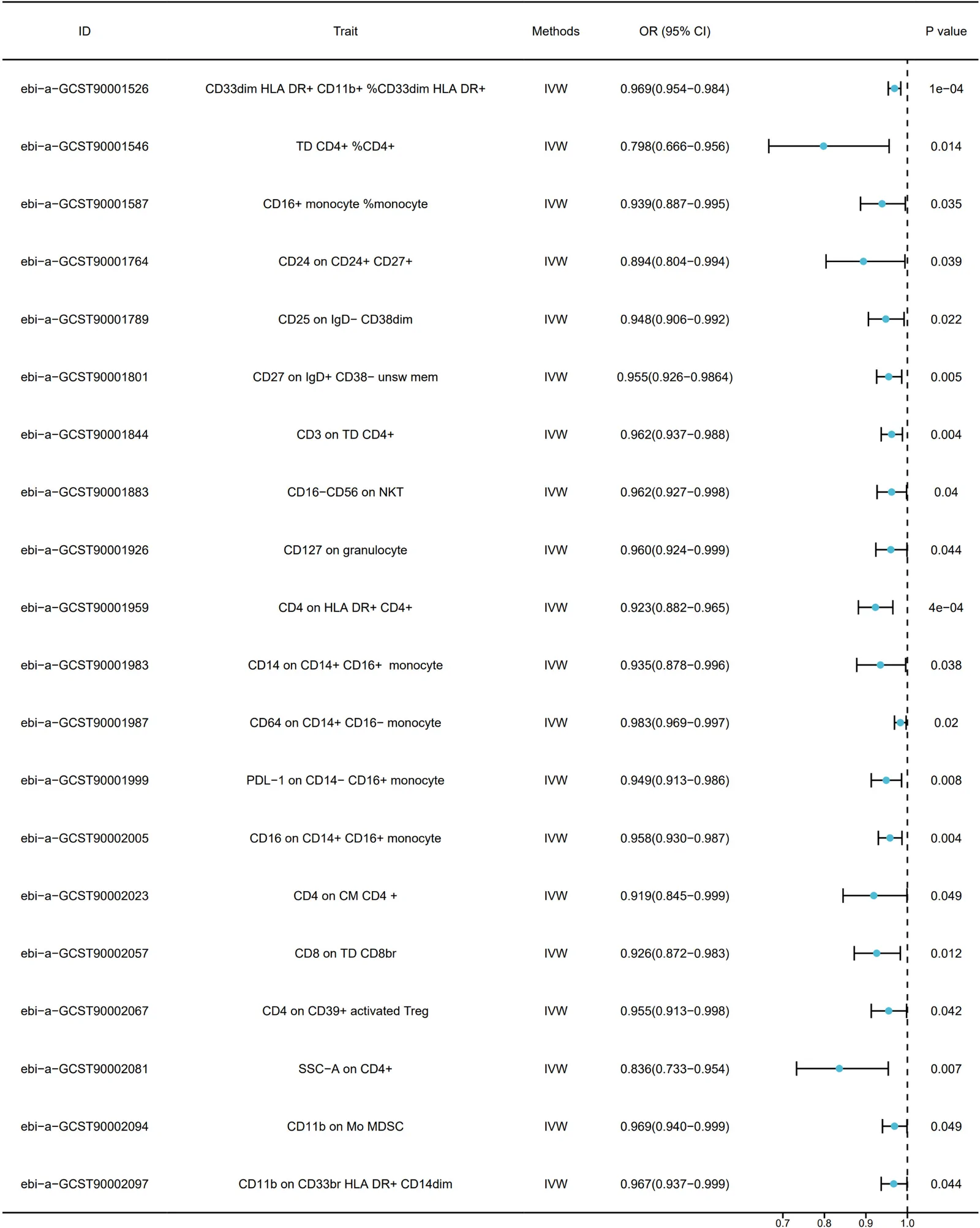
Forest plots showing the causal associations between RA and 20 immune cell traits

For further refinement, we used the odds ratios (ORs) associated with the IVW results to determine which immune cells promoted and protected against RA onset. In general, an OR value > 1 indicates promotion of disease progression, while an OR value < 1 indicates protection and reduced disease incidence. Among the 42 immune cells with positive results, 22 promoted the disease, while 20 protected against it. The 22 immune cells that promoted the disease were distributed across 3 types of B cells, 5 types of CDCs, 6 types of myeloid cells, 2 types of T-cell maturation stages, 1 type of TBNK, 3 types of Treg cells and 2 types of monocytes. Notably, all 5 types of CDCs promoted the disease, suggesting a potentially active role for these cells in promoting disease onset. Most of the trait types observed were MFI, with 14 kinds, while RC had 5 kinds, AC had 3 kinds, and MP had none (Supplementary Table 1 and Fig. 1). The 20 immune cells that protected against the disease were distributed across 3 types of B cells, 0 types of CDCs, 3 types of myeloid cells, 4 types of T-cell maturation stages, 3 types of TBNK cells, 2 types of Treg cells and 5 types of monocytes. Protective immune cells are mainly concentrated in the maturation stages of T cells and monocytes and may have similar functions in defending the body and producing antibodies against foreign substances. Most of the trait types were concentrated in MFI, with 16 kinds, while RC had 3 kinds, AC had 0 kinds, and MP had 1 kind (Supplementary Table 1 and Fig. 2).

The ID numbers of each type of cell and their corresponding immune cells are detailed in the statistical forest plot based on the IVW statistical P value, OR, and 95% confidence intervals (CIs). Based on the IVW statistical P value, we found that the five immune cell types promoting disease were CD8 on CD39+CD8br (Treg Panel) *P* = 1e − 04, OR (95% CI) 1.323 (1.144 − 1.531); HLA DR on plasmacytoid DC (CDC Panel) *P* = 3e − 04, OR (95% CI) 1.229 (1.097 − 1.376); CD33dim HLA DR+CD11b-%CD33dim (Myeloid Cell Panel) *P* = 5e − 04, OR (95% CI) 1.026 (1.011 − 1.041); HLA DR on DC (CDC Panel) *P* = 0.001, OR (95% CI) 1.252 (1.089 − 1.441); and CD11b on basophil (Myeloid Cell Panel) *P* = 0.003, OR (95% CI) 1.050 (1.016 − 1.085) (Supplementary Table 2). Similarly, we observed that the five immune cells with the greatest protection against disease were CD33dim HLA DR+CD11b+ %CD33dim (Myeloid Cell Panel) *P* = 1e − 04, OR (95% CI) 0.969 (0.954 − 0.984); CD4 on HLA DR+CD4+ (TBNK Panel) *P* = 4e − 04, OR (95% CI) 0.923 (0.882 − 0.965); CD3 on TD CD4+ (Maturation stages of T-cells) *P* = 0.004, OR (95% CI) 0.962 (0.937 − 0.988); CD16 on CD14+CD16+ monocytes (Monocyte panel) *P* = 0.004, OR (95% CI) 0.958 (0.930 − 0.987); and CD27 on IgD+ CD38− unsw mem (B-cell panel) *P* = 0.005, OR (95% CI) 0.955 (0.926 − 0.9864) (Supplementary Table 3).

### Investigating the causal relationship between the pathogenesis of RA and immune cells

**Fig. 3:**
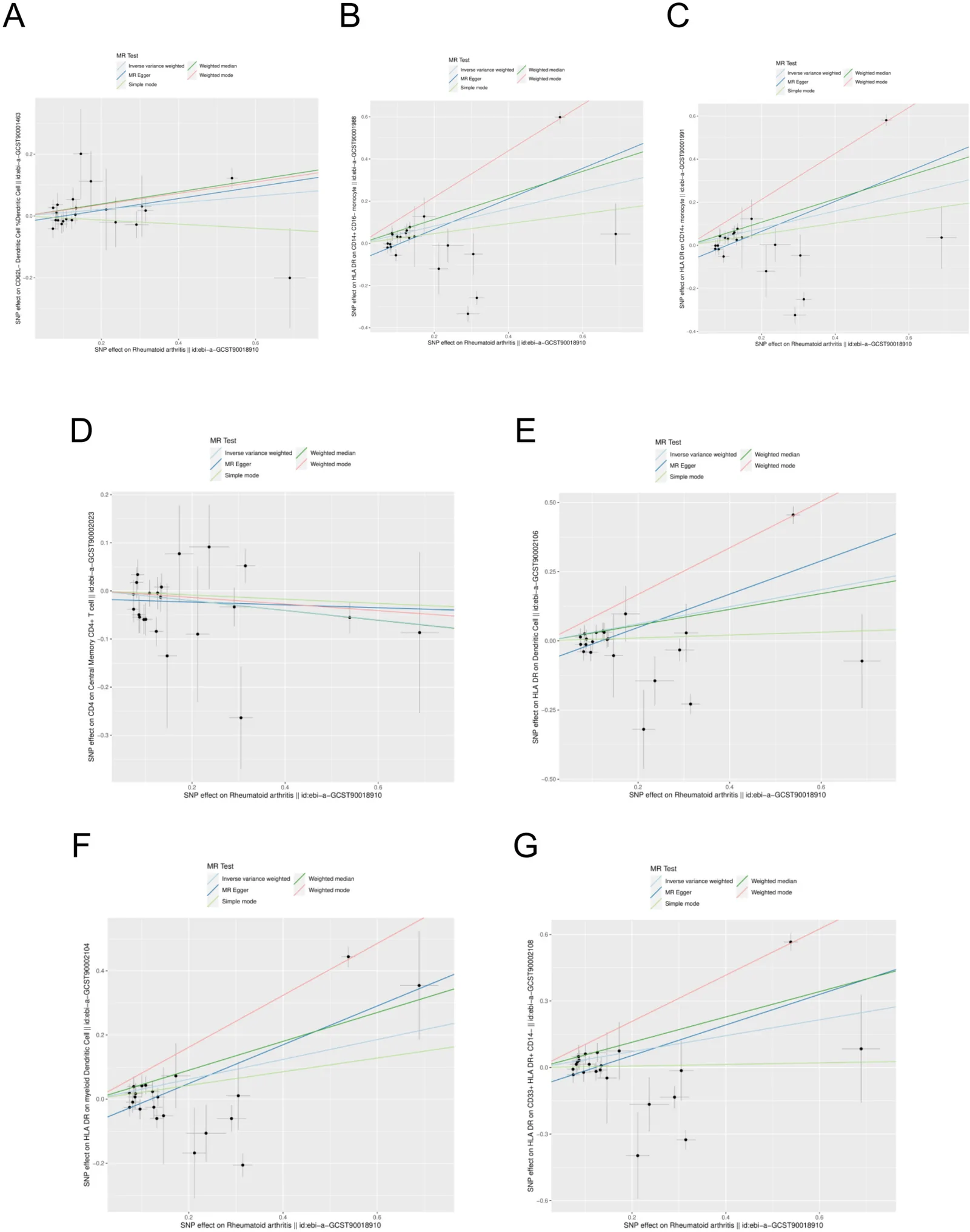
**A**–**G** Causal relationship between RA and 7 immune cell types

**Fig. 4:**
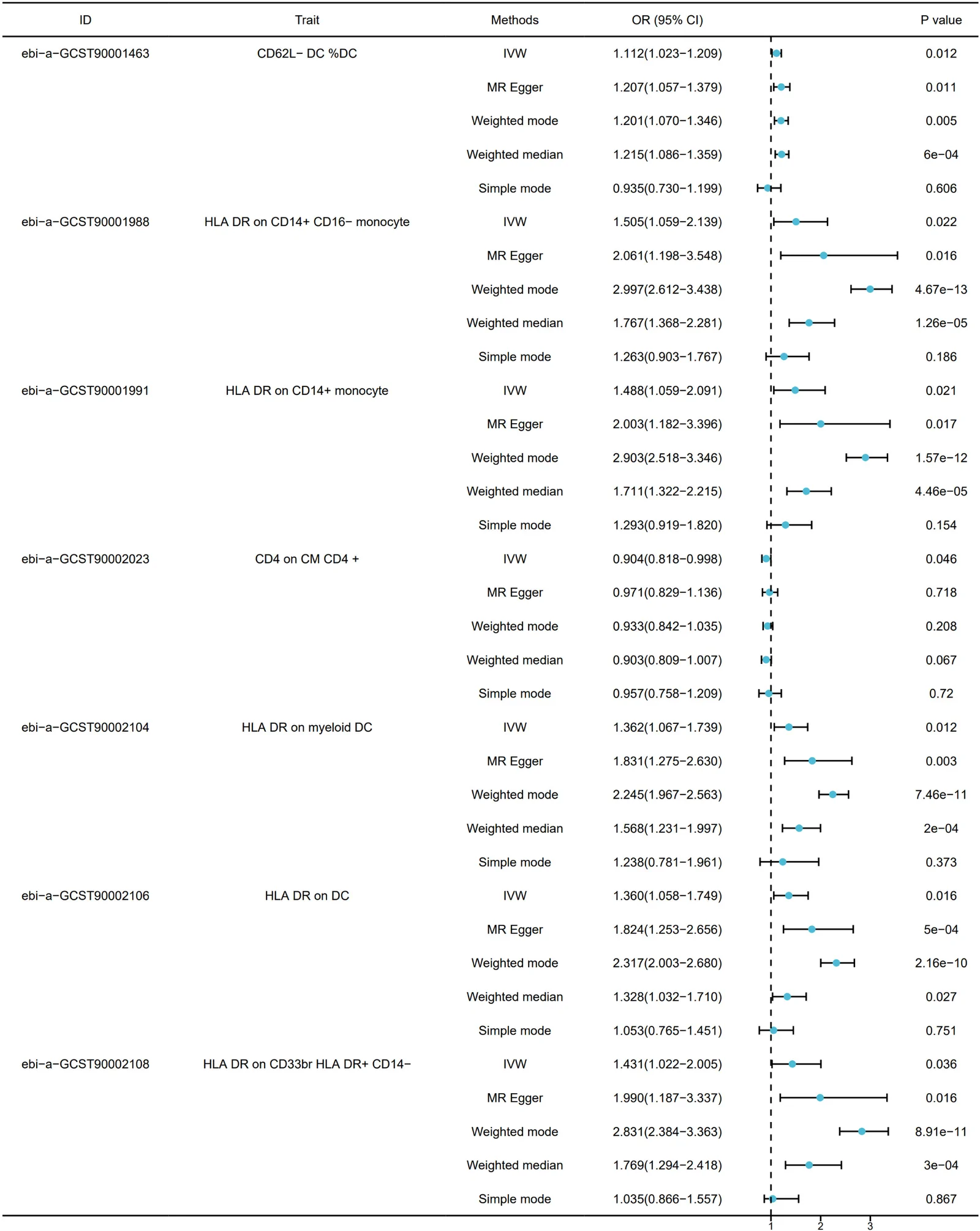
Forest plots showing the causal associations between RA and 7 immune cell traits

To investigate the causal relationship between RA onset and immune cells, we conducted a TSMR analysis using RA as the exposure factor, immune cells with positive results (*n* = 42) as the outcome, and SNPs associated with RA as instrumental variables. The main analytical method used was the IVW method, with a significance threshold set at *P* < 0.05. Using this approach, we identified a causal relationship between RA onset and seven immune cell phenotypes. These included CD62L− DC %DC (cDC Panel), HLA DR on CD14+ CD16− monocyte (Monocyte Panel), HLA DR on CD14+ monocyte (Monocyte Panel), CD4 on CM CD4+ (Maturation stages of T cell), HLA DR on myeloid DC (CDC panel), HLA DR on DC (CDC panel), and HLA DR on CD33br HLA DR+ CD14− (CDC panel). A scatter plot generated through MR analysis visually depicted the causal relationship between RA and these seven immune cells (Fig. 3). Except CD4 on CM CD4+, which showed a protective effect against RA onset, the other six cell types were found to promote RA onset and may serve as key factors, primarily concentrated on the HLA-DR molecule, warranting further attention and discussion. We compared the results of the TSMR with those of five different methods and reached conclusions that were consistent with those of the main IVW analysis (Fig. 4).

To enhance the reliability of our findings, sensitivity analyses were performed. All estimates obtained from the sensitivity analyses aligned with the results of the main IVW analysis, and no significant associations were observed. There was no evidence of heterogeneity in the estimates derived from the genetic instruments. Furthermore, based on the MR‒Egger intercept test, no significant level of pleiotropy was found (Supplementary Table 4).

In conclusion, our TSMR analysis revealed a causal relationship between RA onset and seven immune cell phenotypes. While CD4 on CM CD4+ cells showed a protective effect, the other six cell types were found to promote RA onset, primarily concentrated on the HLA-DR molecule. Our results were consistent across multiple analyses and robust to sensitivity testing, providing strong evidence for the involvement of these immune cells in RA pathogenesis.

## Discussion

In this study, we used bidirectional TwoSampleMR analysis to investigate the causal relationship between RA status and immune cell measurements based on publicly available GWAS data. We first used 731 immune cell phenotypes as exposure factors and RA as the outcome factor. Immune cell phenotype classification was crucial in this study and mainly consisted of four types: AC, MFI reflecting surface antigen levels, MP, and RC. These molecules are typically detected by staining or fluorescence labelling, resulting in measurements of MFI and RC, which represent the density and expression levels of surface markers on immune cells, respectively [31]. Cell population counts play an important role in the immune system, and therefore, their quantity and status are critical for normal immune system function. Cell population counts are typically categorized into RC and AC, where RC indicates the percentage of a specific type of immune cell in the total cell count in a sample, and AC refers to the actual number of a specific type of immune cell in each volume of sample [32]. The activity of immune cells includes their role in the immune response as well as the cytokines and other molecules they produce. These activities can be determined by analysing cytokines secreted by immune cells or cell apoptosis [33]. The morphological features of immune cells, such as size, shape, and colour, are also included. These parameters can be used to monitor morphological changes in different types of immune cells in the immune system and provide useful information for diagnosis and treatment. Morphological parameters include aspects such as cell size, shape, number of cell nuclei, and chromatin morphology, which are usually observed by microscopy and counted manually [34]. By using these immune cell phenotype classifications, we can classify immune cells more precisely and summarize our results more accurately.

We identified 42 immune cell phenotypes that are causally related to RA, with the majority being MFI-based immune cells, totalling 33 different types. The MFI is a measure of the expression level of cell surface markers in immune cell research. By evaluating the fluorescence signal intensity, the MFI reflects the relative abundance of markers and can provide researchers with information on immune status, function, and responsiveness. Comparing the MFI values of different types or subtypes of immune cells can reveal differences in the expression of specific immune-related proteins. For example, in the study of T-cell subsets, comparing the MFI values of specific markers in CD4+ and CD8+ T cells can reveal their different roles and functions in immune responses [35]. In addition, MFI values can also be used to evaluate the function of immune cells. In inflammatory reactions, the expression of certain cell surface receptors may be upregulated, resulting in an increase in the MFI of the corresponding marker [36]. This phenomenon suggests that the cells are in an active immune response state and regulate the expression of markers to achieve their function. By detecting changes in the MFI values of specific markers, we can also evaluate immune cell responsiveness to external stimuli. When immune cells are stimulated by activators, the MFI of specific markers may increase, reflecting the sensitivity and activity of the cells to the stimulus [33]. Analysing the MFI values of specific markers in immune cells can provide important information about their biological characteristics, immune status, function, and responsiveness, which is crucial for understanding the underlying mechanisms of diseases and providing guidance for drug development and immunotherapy.

According to the analysis of 42 positive immune cells, 22 immune cells promoted RA, while 20 immune cells had a protective effect against RA. Through data analysis, it was observed that most of these immune cells belonged to two categories: the CDC group and the myeloid cell group. This finding suggested that these two types of immune cells play crucial roles in promoting the development of RA. CDC group cells, which act as antigen-presenting cells, are capable of capturing and presenting antigens to T cells, thereby initiating a specific immune response. On the other hand, myeloid cells, such as macrophages and neutrophils, are involved in inflammatory responses and joint destruction processes. The interplay between CDC group cells and myeloid cell group cells in RA is complex and diverse. Studies have revealed that in patients with RA, abnormal activation and excessive proliferation of CDC group cells may lead to imbalanced immune responses and tissue damage [37]. Moreover, myeloid cells also participate in the inflammatory response and cytotoxicity in RA, promoting disease progression through the release of inflammatory cytokines and the modulation of T-cell function [38]. Furthermore, the interaction between CDC group cells and myeloid cell group cells may also influence disease development and therapeutic outcomes. In recent years, advanced techniques and model systems have gradually unveiled the molecular mechanisms and interaction networks of CDC group cells and myeloid cell group cells in RA [39]. Therefore, we speculate that the interplay between CDC group cells and myeloid cell group cells in rheumatoid arthritis is intricate and intimate. They participate in the development and progression of RA through pathways involving immune response regulation, the inflammatory response, and joint destruction. Further research will contribute to a deeper understanding of the functions and interaction mechanisms of these cellular components.

In RA, protection against the disease is closely related to the maturation stages of T cells and monocytes, which are two important types of immune cells. T cells are a critical component of the immune system and undergo several key maturation stages, including positive selection, negative selection, and maturation. Abnormalities in T-cell positive and negative selection processes in RA may lead to an increase in autoreactive T cells and attacks on self-tissue. Recent research suggests that therapeutic strategies targeting T cells focus on inhibiting their abnormal activation and proliferation by modulating their signalling pathways and immune checkpoints, such as inhibiting the costimulatory pathway of T-cell activation and regulating T-cell activity through immune checkpoints such as CTLA-4 and PD-1 [40]. Monocytes are an important type of immune cell that differentiates into mature dendritic cells and macrophages in the bone marrow, both of which are involved in regulating immune responses. In RA patients, the activation and differentiation of monocytes may be abnormally stimulated by certain factors. These activated monocytes can produce sufficient levels of inflammatory factors, which can trigger the development and progression of arthritis. Studies have shown that the numbers and activity of mature dendritic cells and macrophages are significantly increased in RA, leading to synovial inflammation and cartilage damage [41]. Therefore, therapeutic strategies targeting monocytes mainly focus on inhibiting monocyte activation and differentiation. TNF-α and IL-6 are important inflammatory factors in RA, and their overexpression can cause abnormal monocyte activation. The inhibition of monocyte activation and differentiation through the regulation of these factors can achieve the goal of treating RA [42]. We speculate that controlling the normal forms of these two types of immune cells and inhibiting their abnormal activation and differentiation are important means of preventing the development of RA, as our research results have verified.

When performing reverse MR, we used RA as the exposure factor and focused on 42 types of immune cells as outcomes. Through our analysis, we found that there is a causal relationship between the onset of RA and the phenotypes of seven immune cells. Of these, RA and six of the cell types had a positive causal relationship, while one cell type had a negative causal relationship. The seven immune cell types we identified through bidirectional TSMR suggest that there is an important connection between the onset of RA and these immune cells. We were surprised to find that the immune cells with a positive causal relationship were concentrated on HLA-DR marker molecules, specifically HLA DR on CD14+ CD16- monocytes, HLA DR on CD14+ monocytes, HLA DR on myeloid DCs, HLA DR on DCs, and HLA DR on CD33br HLA DR+ CD14− immune cells.

HLA-DR is a member of the major histocompatibility complex (MHC) class II family. It is widely expressed on the surfaces of immune cells and plays an important role in regulating immune responses. HLA-DR molecules are closely associated with antigen presentation and T-cell activation. In patients with RA, specific alleles of the HLA-DR gene are closely correlated with susceptibility to RA [43]. HLA-DR molecules present joint-related antigens to CD4+ T cells, activating and perpetuating autoimmune reactions that lead to joint inflammation [44]. Additionally, HLA-DR molecules are involved in regulating the function and activity of immune cells. The synovium of RA patients contains many monocytes and macrophages expressing HLA-DR molecules. These immune cells interact with T cells through the expression of HLA-DR molecules, enhancing inflammatory responses [45]. Furthermore, HLA-DR molecules can regulate antigen presentation by immune cells and promote the production of proinflammatory factors, further exacerbating the development of joint inflammation. Therefore, interfering with the interaction between HLA-DR molecules and T cells can inhibit the inflammatory response and the process of autoimmunity. Other studies have shown the potential therapeutic effects of using antibodies against HLA-DR molecules to block their binding to T cells in the treatment of RA [46]. Further research on HLA-DR molecules is likely to reveal a very important drug target for the treatment of RA, which is consistent with our research results.

The only bidirectional causal association found to have a protective effect was CD4 on CM CD4+ immune cells, which likely implies its protective role in the pathogenesis of RA. CD4 on CM CD4+ cells, as crucial immune protective cells, possess high proliferative capacity and diversity, allowing them to differentiate into various effector T-cell subsets, such as Th1, Th2, and Th17, all of which play important protective roles in RA development. Additionally, CD4 on CM CD4+ cells exhibits memory properties, enabling rapid initiation and generation of more robust and persistent immune responses, which may contribute to RA relapse and progression. However, there remains considerable controversy and ambiguity regarding the specific relationship between CD4 on CM CD4+ cells and RA. Some studies suggest that alterations in the quantity and functionality of CD4 on CM CD4+ cells, such as reduction, abnormal activation, or functional impairment, may be associated with disease progression and severity [47]. Conversely, other studies have proposed the opposite, indicating a protective role of CD4 on CM CD4+ cells in RA, as they are capable of suppressing inflammatory reactions and joint destruction [48]. Nevertheless, our study supports the latter perspective, as we speculate that a comprehensive understanding of the specific mechanisms underlying the role of CD4 on CM CD4+ cells in RA will contribute to a better understanding and treatment of this complex autoimmune joint disorder.

MR is a causal inference method based on genetic instrumental variables that significantly facilitates the discovery of causal relationships between biomarkers and diseases, providing valuable insights for clinical practice. This approach not only supports the identification and validation of biomarkers, the formulation of personalized medical strategies, and the development of new drugs but also contributes to disease prevention, clinical guidelines, public health policies, and optimization of clinical research design, providing more accurate evidence for genetic counselling and thus promoting progress in the medical field and optimization of clinical practice. However, this method also has some limitations. First, GWAS relies on GWAS to identify genetic variants associated with specific exposure factors and is thus limited by the sample size and quality of GWAS. Insufficient or poor-quality data may lead to misidentification of genetic variants [49], thereby affecting the results of MR analysis. In addition, the core assumption of MR analysis is that genetic variants affect disease outcomes only through exposure factors, but there may be unconsidered confounding factors in reality, leading to bias. Moreover, genetic variants may affect disease outcomes through multiple pathways, i.e., through pleiotropy, further complicating causal inference. Moreover, since the impact of individual genetic variants on disease risk is usually small, MR analysis may suffer from insufficient statistical power, potentially resulting in false-negative results. Additionally, MR analysis is typically based on existing datasets, which may not contain information on certain emerging exposure factors, thereby limiting the applicability of the analysis. Furthermore, factors such as linkage disequilibrium and population stratification may also affect the results of MR analysis. Last, the complexity of biological processes may lead to nonlinear relationships between genetic variants and diseases, posing a challenge to MR analysis. Researchers need to exercise caution when interpreting MR analysis results and integrate other research evidence to obtain more reliable causal inferences [50]. To more accurately determine the causal relationship between two variables, multiple methods should be used for cross-validation to comprehensively consider different types of evidence.

## Conclusions

In summary, our study highlights the causal relationship between immune cells and RA through bidirectional two-sample MR analysis, the identification of immune cells causally associated with RA and a summary of our findings. Our study sheds new light on the mechanisms and therapeutic targets of immune cells in RA, providing new directions and clues for further research. However, further studies are needed to fully elucidate the potential mechanisms involved.

## Electronic supplementary material

Below is the link to the electronic supplementary material.

Supplementary Material 1

## Data Availability

There are no new data associated with this article.

## Abbreviations

RA: Rheumatoid arthritis
MR: Mendelian randomization
ILCs: lymphoid cells
RCTs: Randomized controlled trials
TSMR: Two-sample Mendelian randomization
IVW: Inverse-variance weighting
IVs: Instrumental variables
GWAS: Genome-wide association study
ACR: American College of Rheumatology
AC: Absolute cell count
MFI: Median fluorescence intensity
MP: Morphological parameters
RC: Relative cell count
SNPs: Single-nucleotide polymorphisms
LOO: Leave-one-out
MR–PRESSO: MR pleiotropy residual sum and outlier
OR: Odds ratio
CI: Confidence interval
MHC: Major histocompatibility complex

## References

[1] Karras A, Song X, Lin Q. Genomics, transcriptomics and proteomics to elucidate the pathogenesis of rheumatoid arthritis. Rheumatol Int. 2017;37:1257–65. 10.1007/s00296-017-3732-3. 28493174 10.1007/s00296-017-3732-3

[2] Finckh A, Gilbert B, Hodkinson B, et al. Global epidemiology of rheumatoid arthritis. Nat Rev Rheumatol. 2022;18:591–602. 10.1038/s41584-022-00827. 36068354 10.1038/s41584-022-00827-y

[3] Ding Q, Hu W, Wang R, et al. Signaling pathways in rheumatoid arthritis: implications for targeted therapy. Signal Transduc Target Ther. 2013;8:68. 10.1038/s41392-023-01331-9. 10.1038/s41392-023-01331-9 PMC9935929 36797236

[4] Frisell T, Saevarsdottir S, Askling J. Family history of rheumatoid arthritis: an old concept with new developments. Nat Rrev Rheumatol. 2016;12:335–43. 10.1038/nrrheum.2016.52. 10.1038/nrrheum.2016.52 27098907

[5] Chen Y, Wang Y, Jiang X, et al. Dimethylamino group modified polydopamine nanoparticles with positive charges to scavenge cell-free DNA for rheumatoid arthritis therapy. Bioac Mater. 2022;18:409–20. 10.1016/j.bioactmat.2022.03.028. 10.1016/j.bioactmat.2022.03.028 PMC8968194 35415310

[6] Smith MH, Gao VR, Periyakoil PK, et al. Drivers of heterogeneity in synovial fibroblasts in rheumatoid arthritis. Nat Immunol. 2023;24:1200–10. 10.1038/s41590-023-01527-9. 37277655 10.1038/s41590-023-01527-9 PMC10307631

[7] Komatsu N, Takayanagi H. Mechanisms of joint destruction in rheumatoid arthritis - immune cell-fibroblast-bone interactions. Nat Rev Rheumat. 2022;18:415–29. 10.1038/s41584-022-00793-5. 10.1038/s41584-022-00793-5 35705856

[8] Yamada S, Nagafuchi Y, Wang M, et al. Immunomics analysis of rheumatoid arthritis identified precursor dendritic cells as a key cell subset of treatment resistance. Ann Rheum Dis. 2023;82:809–19. 10.1136/ard-2022-223645. 36918189 10.1136/ard-2022-223645 PMC10314026

[9] Barrat FJ, Crow MK, Ivashkiv LB. Interferon target-gene expression and epigenomic signatures in health and disease. Nat Immunol. 2019;20:1574–83. 10.1038/s41590-019-0466-2. 31745335 10.1038/s41590-019-0466-2 PMC7024546

[10] Murdaca G, Colombo BM, Puppo F. The role of Th17 lymphocytes in the autoimmune and chronic inflammatory diseases. Intern Emerg Med. 2011;6:487–95. 10.1007/s11739-011-0517-7. 21258875 10.1007/s11739-011-0517-7

[11] Tett A, Pasolli E, Masetti G, et al. Prevotella diversity, niches and interactions with the human host. Nat Rev Microbiol. 2021;19:585–99. 10.1038/s41579-021-00559-y. 34050328 10.1038/s41579-021-00559-y PMC11290707

[12] Kang J, Eun Y, Jang W, et al. Rheumatoid arthritis and risk of parkinson disease in Korea. JAMA Neurol. 2023;80:634–41. 10.1001/jamaneurol.2023.0932. 37126341 10.1001/jamaneurol.2023.0932 PMC10152376

[13] Brenowitz WD, Yaffe K. Observational studies in Alzheimer disease: bridging preclinical studies and clinical trials. Nat Rev Neurol. 2022;18:747–57. 10.1038/s41582-022-00733-7. 36316487 10.1038/s41582-022-00733-7 PMC9894623

[14] Yu N, Qi H, Guo Y, et al. Associations between rheumatoid arthritis and skin cancer: a bidirectional two-sample Mendelian randomization study. J Am Acad Dermatol. 2023;23:02862–1. 10.1016/j.jaad.2023.09.046. 10.1016/j.jaad.2023.09.046 37758025

[15] Qin C, Diaz-Gallo LM, Tang B, et al. Repurposing antidiabetic drugs for rheumatoid arthritis: results from a two-sample Mendelian randomization study. Eur J Epidemiol. 2023;38:809–19. 10.1007/s10654-023-01000-9. 37052755 10.1007/s10654-023-01000-9 PMC10276071

[16] Burgess S, Butterworth A, Thompson SG. Mendelian randomization analysis with multiple genetic variants using summarized data. Genet Epidemiol. 2013;37:658–65. 10.1002/gepi.21758. 24114802 10.1002/gepi.21758 PMC4377079

[17] Sakaue S, Kanai M, Tanigawa Y, et al. A cross-population atlas of genetic associations for 220 human phenotypes. Nat Genet. 2021;53:1415–24. 10.1038/s41588-021-00931-x. 34594039 10.1038/s41588-021-00931-x PMC12208603

[18] Stahl EA, Raychaudhuri S, Remmers EF, et al. Genome-wide association study meta-analysis identifies seven new rheumatoid arthritis risk loci. Nat Genet. 2010;42:508–14. 10.1038/ng.582. 20453842 10.1038/ng.582 PMC4243840

[19] Bowden J, Davey SG, Burgess S. Mendelian randomization with invalid instruments: effect estimation and bias detection through Egger regression. Int J Epidemiol. 2015;44:512–25. 10.1093/ije/dyv080. 26050253 10.1093/ije/dyv080 PMC4469799

[20] Orrù V, Steri M, Sidore C, et al. Complex genetic signatures in immune cells underlie autoimmunity and inform therapy. Nat Genet. 2020;52:1036–45. 10.1038/s41588-020-0684-4. 32929287 10.1038/s41588-020-0684-4 PMC8517961

[21] Burgess S, Butterworth A, Thompson SG. Mendelian randomization analysis with multiple genetic variants using summarized data. Genet Epidemiol. 2013;37:658–65. 10.1002/gepi.21758. PMID:24114802. 24114802 10.1002/gepi.21758 PMC4377079

[22] Sang N, Gao RC, Zhang MY et al. Causal relationship between sleep traits and risk of systemic lupus erythematosus: a two-sample Mendelian randomization study. Front Immunol. 2022:13:918749. 10.3389/fimmu.2022.918749 10.3389/fimmu.2022.918749 PMC9248809 35784289

[23] Huang S, Tian F, Yang X, et al. Physical activity and systemic lupus erythematosus among European populations: a two-sample mendelian randomization study. Front Genet. 2022;12:784922. 10.3389/fgene.2021.784922. 35211151 10.3389/fgene.2021.784922 PMC8861300

[24] Sidore C, Busonero F, Maschio A, et al. Genome sequencing elucidates Sardinian genetic architecture and augments association analyses for lipid and blood inflammatory markers. Nat Genet. 2015;47:1272–81. 10.1038/ng.3368. 26366554 10.1038/ng.3368 PMC4627508

[25] Bowden J, Davey SG, Haycock PC, et al. Consistent estimation in Mendelian randomization with some invalid instruments using a weighted median estimator. Genet Epidemiol. 2016;40:304–14. 10.1002/gepi.21965. 27061298 10.1002/gepi.21965 PMC4849733

[26] Burgess S, Thompson SG. Interpreting findings from Mendelian randomization using the MR-egger method. Eur J Epidemiol. 2017;32:377–89. 10.1007/s10654-017-0255-x. 28527048 10.1007/s10654-017-0255-x PMC5506233

[27] Xiang K, Wang P, Xu Z, et al. Causal effects of gut microbiome on systemic lupus erythematosus: a two-sample Mendelian randomization study. Front Immunol. 2021;12:667097. 10.3389/fimmu.2021.667097. 34557183 10.3389/fimmu.2021.667097 PMC8453215

[28] Sun W, Zhang L, Liu W, et al. Stroke and myocardial infarction: a bidirectional mendelian randomization study. Int J GenMed. 2021;14:9537–45. 10.2147/IJGM.S337681. 10.2147/IJGM.S337681 PMC8670204 34916835

[29] Hemani G, Zheng J, Elsworth B, et al. The MR-base platform supports systematic causal inference across the human phenome. Elife. 2018;7:e34408. 10.7554/eLife.34408. 29846171 10.7554/eLife.34408 PMC5976434

[30] Bae SC, Lee YH. Vitamin d level and risk of systemic lupus erythematosus and rheumatoid arthritis: a Mendelian randomization. Clin Rheumatol. 2018;37:2415–21. 10.1007/s10067-018-4152-9. 29799605 10.1007/s10067-018-4152-9

[31] Lamers-Kok N, Panella D, Georgoudaki AM, et al. Natural killer cells in clinical development as non-engineered, engineered, and combination therapies. J Hematol Oncol. 2022;15:164. 10.1186/s13045-022-01382-5. 36348457 10.1186/s13045-022-01382-5 PMC9644572

[32] Chen MH, Raffield LM, Mousas A, et al. Trans-ethnic and ancestry-specific blood-cell genetics in 746,667 individuals from 5 global populations. Cell. 2020;182:1198–213. 10.1016/j.cell.2020.06.045. 32888493 10.1016/j.cell.2020.06.045 PMC7480402

[33] Soerens AG, Künzli M, Quarnstrom CF, et al. Functional T cells are capable of supernumerary cell division and longevity. Nature. 2023;614:762–6. 10.1038/s41586-022-05626-9. 36653453 10.1038/s41586-022-05626-9 PMC11617068

[34] Johnson AM, Boland JM, Wrobel J, et al. Cancer cell-specific major histocompatibility complex II expression as a determinant of the immune infiltrate organization and function in the NSCLC tumor microenvironment. J Thor Oncol. 2021;16:1694–704. 10.1016/j.jtho.2021.05.004. 10.1016/j.jtho.2021.05.004 PMC8464501 34048945

[35] Paul MS, Pamela SO. The roles of CD8+ T cell subsets in antitumor immunity. Trends Cell Biol. 2020;30:695–704. 10.1016/j.tcb.2020.06.003. 32624246 10.1016/j.tcb.2020.06.003

[36] Chen T, Guo J, Cai Z, et al. Th9 cell differentiation and its dual effects in tumor development. Front Immunol. 2020;11:1026. 10.3389/fimmu.2020.01026. 32508847 10.3389/fimmu.2020.01026 PMC7251969

[37] Makkar RR, Kereiakes DJ, Aguirre F, et al. Intracoronary ALLogeneic heart STem cells to achieve myocardial regeneration (ALLSTAR): a randomized, placebo-controlled, double-blinded trial. Eur Heart J. 2020;41:3451–8. 10.1093/eurheartj/ehaa541. 32749459 10.1093/eurheartj/ehaa541

[38] Kurowska SM, Alivernini S. Synovial tissue macrophages in joint homeostasis, rheumatoid arthritis and disease remission. Nat Rev Rheumatol. 2022;18:384–97. 10.1038/s41584-022-00790-8. 35672464 10.1038/s41584-022-00790-8

[39] Delgado AC, Calvet MM, Triguero MA, et al. NLRC4-mediated activation of CD1c+ DC contributes to perpetuation of synovitis in rheumatoid arthritis. JCI Insight. 2022;7:e152886. 10.1172/jci.insight.152886. 36194479 10.1172/jci.insight.152886 PMC9746818

[40] Qin S, Xu L, Yi M, et al. Novel immune checkpoint targets: moving beyond PD-1 and CTLA-4. Mol Cancer. 2019;18:155. 10.1186/s12943-019-1091-2. 31690319 10.1186/s12943-019-1091-2 PMC6833286

[41] Mulder K, Patel AA, Kong WT, et al. Cross-tissue single-cell landscape of human monocytes and macrophages in health and disease. Immunity. 2021;54:1883–e19005. 10.1016/j.immuni.2021.07.007. 34331874 10.1016/j.immuni.2021.07.007

[42] Yokota K, Sato K, Miyazaki T, et al. Characterization and function of tumor necrosis factor and interleukin-6-induced osteoclasts in rheumatoid arthritis. Arthritis Rheumatol. 2021;73:1145–54. 10.1002/art.41666. 33512089 10.1002/art.41666 PMC8361923

[43] Maggi J, Carrascal M, Soto L, et al. Isolation of HLA-DR-naturally presented peptides identifies T-cell epitopes for rheumatoid arthritis. Ann Rheum Dis. 2022;81:1096–105. 10.1136/annrheumdis-2021-220371. 35459695 10.1136/annrheumdis-2021-220371

[44] Turcinov S, Af KE, Van SB, et al. Diversity and clonality of T cell receptor repertoire and antigen specificities in small joints of early rheumatoid arthritis. Arthritis Rheumatol. 2023;75:673–84. 10.1002/art.42407. 36409582 10.1002/art.42407

[45] Kanaan SB, Sensoy O, Yan Z, et al. Immunogenicity of a rheumatoid arthritis protective sequence when acquired through microchimerism. Pro Nat Acad Sci U S A. 2019;116:19600–8. 10.1073/pnas.1904779116. 10.1073/pnas.1904779116 PMC6765309 31501349

[46] Wu X, Liu Y, Jin S, et al. Single-cell sequencing of immune cells from anticitrullinated peptide antibody positive and negative rheumatoid arthritis. Nat Commun. 2021;12:4977. 10.1038/s41467-021-25246-7. 34404786 10.1038/s41467-021-25246-7 PMC8371160

[47] Floudas A, Neto N, Orr C, et al. Loss of balance between protective and pro-inflammatory synovial tissue T-cell polyfunctionality predates clinical onset of rheumatoid arthritis. Ann Rheum Dis. 2022;81:193–205. 10.1136/annrheumdis-2021-220458. 34598926 10.1136/annrheumdis-2021-220458

[48] Keller CW, Adamopoulos IE, Lünemann JD. Autophagy pathways in autoimmune diseases. J Autoimmun. 2023;136:103030. 10.1016/j.jaut.2023.103030. 37001435 10.1016/j.jaut.2023.103030 PMC10709713

[49] Smith GD, Ebrahim S. Mendelian randomization’: can genetic epidemiology contribute to understanding environmental determinants of disease? Int J epidemiol. 2003;32:1–22. 10.1093/ije/dyg070. 12689998 10.1093/ije/dyg070

[50] Lawlor DA, Harbord RM, Sterne JA, et al. Mendelian randomization: using genes as instruments for making causal inferences in epidemiology. Stat Med. 2008;27:1133–63. 10.1002/sim.3034. 17886233 10.1002/sim.3034

