# Supplementary material for: Analysis of the causal relationship between immune cells and rheumatoid arthritis from the perspective of genetic variation: a bidirectional two-sample Mendelian randomization study
Source: Adv Rheumatol. 2024 Nov 1;64:83. doi: 10.1186/s42358-024-00425-4 (PMC13488670; doi:10.1186/s42358-024-00425-4)
Supplement: Supplementary file 1 [file 42358_2024_425_MOESM1_ESM.doc]

**Additional file 1:**

**Supplementary Table 1.** The ID, P value, trait type, panel, statistical trait name and trait of 42 immune cells.

**Supplementary Table 2.** The trait, panel, P value, and OR (95% CI) information for the top five disease-promoting immune cells

**Supplementary Table 3.** The trait, panel, P value, and OR (95% CI) information for the top five immune cells involved in disease protection.

**Supplementary Table 4.** The MR sensitivity of 7 immune cell types was analysed as an outcome variable, with RA as the exposure factor.

**Supplementary Table 5.** Details of sensitivity analysis of MR Results of 22 immune cells and RA。

**Supplementary Table 6.** Details of sensitivity analysis of MR Results of 20 immune cells and RA.

**Supplement Table 7.** SNPs used in forward MR analysis.

**Supplementary Figure S1.** A-G LOO diagram of 7 immune cells.

**Supplementary Figure S2.** MR Plots for Relationship of 22 immune cells with RA.
